# Isolation Hospitals as Centres of Infection

**Published:** 1895-02-23

**Authors:** 


### The Hospital

An Institutional Journal of

The Medical Sciences and Hospital Administration, with

AN EXTRA NURSING SUPPLEMENT.

Vol. XVII.—No. 439.] [Feb. 23, 1895.

### Isolation Hospitals as Centres of Infection.

A well informed medical practitioner recently challenged the medical profession and the public with this contention that, notwithstanding the compulsory notification of infectious diseases and the excellent provision of isolation hospitals within the metropolitan area, there has been no diminution in the frequency or severity of epidemics of infectious diseases, and no lessening of mortality among those attacked. The meaning of this, in plain English, is that in spite of all our costly efforts to suppress or, at least, to limit the spread and the severity of infectious diseases, we have not only not been largely successful, but we have accomplished that which is equivalent to nothing at all. This contention we do not believe to be justified by facts. On the contrary, it can hardly be doubted that notification and isolation have both rendered signal service to the public, and that these means tend in the direction of ultimate relative, if not of absolute, suppression of diseases of the infectious order.

But though we cannot endorse the contention of the pessimistic correspondent of the *Times* as to the entire uselessness of notification and isolation, we can admit that there is much significance in what he affirms. His contention may be deferred to so far as this, that notification and isolation have, to a certain extent, disappointed their sponsors and admirers. But if so much be admitted, what then? Why have notification and isolation disappointed expectation? And this further question, Are the principles of notification and isolation in themselves unsound, or are not the conditions under which these principles have been carried into effect the causes of the comparative failure we have to deplore?

In the first place, it is clear that notification by itself can do nothing at all towards the suppression of epidemics. Notification is merely the first step in the process of isolation. It is isolation to which we must look for success. If, when isolation shall have been carried out in all its details, we still find that no impression is made upon the spread of infectious diseases, then may the sanitarian almost throw up his hands in despair.

The eye of practical science must be directed to isolation hospitals in this connection. Have isolation hospitals failed? Why have they failed? Are they themselves sources of danger? If the last of these three interrogations be answered in the affirmative, we have at once at least a partial explanation of the failure of the whole system of notification and isolation. It is to be confessed, it has already been confessed both by Royal Commission and the sanitary authorities of the Local Government Board, that, at any rate in some instances, isolation hospitals have proved themselves centres of infection. Let us take the case of small-pox hospitals. "Ever since the issue in 1882 of the Report of the Royal Commission on Small-pox and Fever Hospitals," says Dr. Thorne Thorne, "great difficulty has arisen in the selection of sites for the reception of patients suffering from small-pox. Small-pox hospitals have again and again served to disseminate that disease to neighbouring communities." Here we are face to face with hard facts, facts proved and admitted in the very quarter where the admission is most significant and most likely to be productive of good results. The sanitary authorities of the Local Government Board, though they must have found the potion a most nauseous one to swallow, have yet swallowed it with most honourable scientific loyalty, and now both they and the public are waiting to see the effects of the stimulating draught.

If it be true, as it is, that small-pox hospitals have proved themselves centres of infection, may it not also be true, though the fact is not yet proved, and may never be proved—may it not, nevertheless, be true that isolation hospitals for other infectious diseases are also centres of infection?

Some practical questions of the first order of importance arise here. Isolation hospitals tend to be large. Perhaps they ought to be small. They are kept in many instances in practically continuous use. Perhaps they ought to be disused periodically; and perhaps, also, they ought to be unroofed, or even unwooded altogether, and their permanent portions purified by fire. In any case, and whilst fuller investigations are being made, we cannot, without a criminal disregard of neighbouring populations, insist upon building any more small-pox hospitals in near contiguity to populations larger than those of mere hamlets. Possibly also hospitals for other infectious diseases, unless it be in the doubtful case of typhoid, should be located at much greater distances from considerable populations than we have hitherto thought it to be either necessary or expedient to place them. There is also another and a very important aspect of the question. More knowledge of bacteriology, and especially of the life conditions of specific bacteria is imperatively demanded. How do pathogenic bacteria pass from person to person? Since these organisms differ in form and in other vital conditions, it is obvious that they may differ also in their power and mode of transference from place to place and from person to person. A full knowledge of all these conditions of bacterial life and activity would throw light of the most important character upon those conditions which are indispensable to safety in the location and construction of isolation hospitals. The whole subject is one of the profoundest importance to the inhabitants of a densely-peopled country like our own; and it is but too evident that our knowledge of the indispensable conditions of safety for both the sick and the well in this connection is still far from perfect.

